# Evaluation of 1021Bp, a close relative of *Pseudomonas eucalypticola*, for potential of plant growth promotion, fungal pathogen suppression and boxwood blight control

**DOI:** 10.1186/s12866-024-03497-w

**Published:** 2024-09-14

**Authors:** Ping Kong, Chuanxue Hong

**Affiliations:** Hampton Roads Agricultural Research and Extension Center, Virginia Tech, Virginia Beach, USA

**Keywords:** Antifungal activity, Boxwood, Boxwood blight control, Leaf endophyte, Tween 20, Plant growth promotion trait, *P. fluorescens*

## Abstract

**Background:**

*Pseudomonas eucalypticola*, a new species of the *P. fluorescens* group that generates most *Pseudomonas*-based biocontrol agents, has not been found in any plants other than *Eucalyptus dunnii* leaves. Except for antagonism to the growth of a few fungi, its features in plant growth promotion and disease control have not been evaluated. Here, we identified a similar species of *P. eucalypticola*, 1021Bp, from endophyte cultures of healthy leaves of English boxwood (*Buxus sempervirens* ‘Suffruticosa’) and investigated its antifungal activity, plant growth promotion traits, and potential for boxwood blight control.

**Results:**

Colorimetric or plate assays showed the properties of 1021Bp in nitrogen fixation, phosphate solubilization, and production of indole-3-acetic acid (IAA) and siderophores, as well as the growth suppression of all five plant fungal pathogens, including causal agents of widespread plant diseases, gray mold, and anthracnose. Boxwood plant leaves received 87.4% and 65.8% protection from infection when sprayed with cell-free cultural supernatant (CFS) but not the resuspended bacterial cells at 10^8–9^/mL of 1021Bp at one and seven days before inoculation (dbi) with boxwood blight pathogen, *Calonectria pseudonaviculata*, at 5 × 10^4^ spores/mL. They also received similarly high protection with the 1021Bp cell culture without separation of cells and CFS at 14 dbi (67.5%), suggesting a key role of 1021Bp metabolites in disease control.

**Conclusions:**

Given the features of plant growth and health and its similarity to *P. eucalypticola* with the *P. fluorescens* lineage, 1021Bp has great potential to be developed as a safe and environmentally friendly biofungicide and biofertilizer. However, its metabolites are the major contributors to 1021Bp activity for plant growth and health. Application with the bacterial cells alone, especially with nonionic surfactants, may result in poor performance unless survival conditions are present.

**Supplementary Information:**

The online version contains supplementary material available at 10.1186/s12866-024-03497-w.

## Background

Boxwood blight caused by *Calonectria* species *C. pseudonaviculata* (*Cps*) or/and *C. henricotiae* (*Che*) is the most serious disease of boxwood (*Buxus* sp.), which has disfigured many precious public and private gardens and landscapes worldwide since it emerged three decades ago [1, 2]. The symptoms of the disease include leaf spots or blotches, rapid defoliation, and stem lesions [3]. Control of this disease relies on the application of fungicides [4]. Unfortunately, among the few registered and most adopted fungicides for disease control, chrolorothalonil has been declared a major carcinogen (European Directive 2009/128/EC on the “Sustainable Use of Pesticides”). Intensive applications of fungicides may soon result in chemical resistance, as has been shown in other calonectria diseases of ornamental crops [5]. Alternative control measures are urgently needed to mitigate the health and environmental hazards and fungicide resistance risks.

Selecting and using tolerant cultivars, good cultural practices and identifying effective biocontrol agents have received increased attention in boxwood blight management [3, 6, 7]. Among the identified biocontrol agents for boxwood blight [8–11], *Pseudomonas* species accounts for the most. They were isolated either from irrigation water or boxwood leaves. However, all of them are relatively less effective compared to the boxwood leaf endophyte *Burkholderia* sp. SSG [12], which mainly depends on cells for efficacy [8]. Meanwhile, because it is a member of the *B. cepacia* complex (Bcc), a group of *Burkholderia* with medical concerns [13], its application is likely delayed due to registration difficulties, despite the fact that SSG originated from plants and is negative for Bcc epidemic strain marker [8, 14]. The good news is that metabolites from biocontrol agents have been a focus for developing fungicides of microbial origin because they can be synthesized biologically, and the products may be not only specifically effective on the target organisms but also inherently biodegradable [15]. Thus, to safeguard plants and sustain a healthy and safe ecosystem, identifying agents that use metabolites as a major workforce is urgently needed for rapidly developing such biofungicides.

Bacterial endophytes in healthy boxwood have been associated with plant tolerance [16, 17], and a couple of them have been identified as potential biocontrol agents [9, 18]. In endophytic culture plates with leaf tissue extracts of a 10-year-old healthy boxwood, we isolated a bacterium, 1021Bp that was similar in 16 S rRNA gene sequence to *Pseudomonas eucalypticola* NP-1 that was originally from *Eucalyptus dunnii* leaves and belongs to the *P. fluorescens* lineage [19]. In this lineage, bacteria are aerobic and Gram-negative. Some of them have been used as biocontrol agents because of their rare linkage to human diseases and promising antifungal and/or plant growth promotion traits [20–23]. Although *P. fluorescens* is ubiquitously present in agricultural soils and well adapted to grow in the rhizosphere, certain strains may have an endophytic lifestyle in plants [24]. *P. eucalypticola* is likely one of the latter. However, its efficacy in plant disease control and plant growth-promoting properties have not been reported.

This study was to characterize 1021Bp including determining its in-vitro activities in suppressing *Cps* and other important plant fungal pathogens and traits in promoting plant growth and evaluating its *in-planta* efficacy on boxwood blight control with cell culture, resuspended cells, and cell metabolites. The goal was to make the most of boxwood plant endophyte collection and identify a SSG substitute that possesses not only both features of biofungicides and biofertilizers but also less environmental concerns to safely promote plant yield and health.

## Methods

### 1021Bp isolation

1021Bp used for this study was subcultured from an isolation plate spread with the preparation of three randomly selected leaves of English boxwood (*Buxus sempervirens* ‘Suffruticosa’) after surface sterilization. The leaves were taken from 10-year-old healthy plants in a public garden to study cultural endophyte communities [16]. Cultural endophytes were obtained as described previously. Briefly, the leaves were surface sterilized with 70% ethanol and 10% bleach, then rinsed three times in a large volume of sterilized distilled water (SDW) to remove epiphytes and the associated DNA residues. The sterilized leaves were then cut into small pieces and placed in a presterilized 2 mL microtube containing 1 mL SDW, 0.3 mL 1.4 mm Zirconium beads (MP Biomedicals, Santa Ana, CA, USA), and a 0.35 mm ceramic sphere (OPS Diagnostics, Lebanon, NJ, USA) and homogenized for 3 × 30 s at speed 6 on the FastPrep-24™ Classic bead beating grinder and lysis system (MP Biomedicals, Santa Ana, CA, USA). To grow the microbes, a 0.3-mL aliquot of the resultant suspension was plated on potato dextrose agar (PDA, Sigma-Aldrich, St. Louis, MO, USA) in each 9-cm replicate Petri dish. One of two tan colonies (Additional file 1) named 1021Bp was isolated after 3 days at 23 °C and grown on PDA at 28 °C for 48 h in an incubator (Percival, Boone, IA, USA). To store the culture, its single colonies were grown in Difco™ nutrient broth (NB, Becton, Dickinson, and Company, Sparks, MD, USA) on a shaker (New Brunswick Scientific Inc., Edison, NJ, USA) at 180 rpm overnight, and the culture with a 50% glycerol solution was placed in a -80 °C freezer until use.

### 1021Bp liquid culture, cell suspension, and cell-free supernatant (CFS)

To test 1021Bp in vitro and *in planta* activities, 1021Bp was retrieved from storage and grown on PDA plates. For liquid culture, a single colony from the plate was placed in a 5-mL NB, and shaken at 180 rpm at 28 °C overnight to make a seed culture, and 1 mL of the seed culture was transferred into 100 mL NB and incubated for an additional 18 h or until OD600 = 0.6 to 0.8 under the same conditions to obtain 10^8^ to 10^9^ colony-forming units (CFU) per mL (UFC/mL). To separate the cells and CFS in the culture, the liquid culture was centrifuged at 14,210× g for 15 min with a Sorvall^®^ RC 5 C Plus Superspeed Centrifuge (Mashall Scientific, NH, USA). The supernatant that passed through a 0.22-µm Stericup^®^ and Steritop^®^ Vacuum Driven Disposable Bottle Top Filter (EMD Millipore, MA, USA) was used as CFS. The pellet of bacterial cells was resuspended in 0.01% Tween 20 (Sigma-Aldrich, MO, USA).

### Analyses of plant growth promotion (PGP) traits

PGP characters of 1021Bp, including nitrogen fixation, phosphate solubilization, siderophore, and indole-3-acetic acid (IAA) production, were analyzed as described previously for the boxwood bacterial endophyte *Burkholderia* sp. SSG [25]. Briefly, IAA production was measured using the colorimetric method [26] with a minor modification as described previously for SSG [25]. The nitrogen fixation ability of 1021Bp was determined by growing the bacterium on the nitrogen-free agar medium for 4 days as described previously (Liaqat and Eltem, 2016).

The ability of 1021Bp to solubilize phosphate was determined qualitatively and quantitatively with the National Botanical Research Institute’s Phosphate (NBRIP) agar and broth medium [27, 28], with minor modifications as described previously for SSG [25]. Specifically, in the qualitative assay, a 10-µL aliquot of the bacterial seed culture was pipetted onto each of three sterilized Whatman filter paper disks that were placed on NBRIP agar at the points of an equilateral triangle and checked for inducing halo development around the disks after 7 days of incubation at 27 °C. In the quantitative assay, 0.3 mL of an overnight NB culture of 1021Bp was added to 30-mL NBRIP broth containing 150 mg Ca_3_(PO4)_2_ as an insoluble form of phosphate. After shaking at 27 °C for 7 days, the mix was centrifuged at 13,416 g for 10 min to obtain the supernatant to quantify soluble phosphate by the bacterium. For measurement, the supernatant was autoclaved for 20 min at 4 °C, and 1 mL of the supernatant or its dilution was added to 2 mL of 2.5% ammonium molybdate and 0.5 mL of 10 mol/L sulfuric acid, mixed with 1 mL of 0.5 mol/L hydrazine hydrate solution, then brought to 25 mL with SDW. An aliquot of the diluted mix was blanked with the control solution without the bacterium and measured for absorbance at 840 nm on a DU800^®^ spectrophotometer (Beckman Coulter, CA, USA).

Siderophore production by 1021Bp was determined by streaking the bacterium on the blue agar medium containing chrome azurol S with an indicator hexadecyltrimethylammonium bromide and observing color change after 48 h at 28 °C [29]. All the assays included three replicate plates and were done twice.

### Evaluation of 1021Bp for fungal pathogen growth suppression

Five important fungal plant pathogens collected from Virginia, USA (Table 1) were tested in the dual culture assay on PDA plates, as described previously [18]. A mycelial plug from the edge of one week-old culture of a target fungus was placed in the center of each of three 90-mm PDA plates and an 18 h-old liquid culture of 1021Bp was streaked equidistantly on the sides of the plug. Another set of three plates with fungal plugs was streaked with NB as controls. All the plates were incubated at 25 °C and checked for fungal growth after 5 and 10 days. The diameters of fungal growth in the control plates without the bacterium were compared with those cocultured with the bacterium to calculate the suppression of fungal growth by 1021Bp. Two independent experiments were performed.

**Table 1:** Test fungal pathogens for growth suppression by 1021Bp in the dual culture assay

Pathogen	Source	Host	Disease
*Alternaria tenuissima*	Pansy	Blueberries, tomatoes, grapevine, and strawberries	Leaf spot and blight
*Botrytis cinerea*	Pansy	Coreopsis, dianthus, heuchera, lavender, rudbeckia, wine grapes, strawberries, leafy vegetables	Gray mold
*Calonectria pseudonaviculata*	Boxwood	Boxwood, sweet box, pachysandra	Blight, stem streaks
*Colletotrichum gloeosporioides*	Hydrangea	Citrus, yam, papaya, avocado, coffee, eggplant, sweet pepper, and tomato	Anthracnose
*Pseudonectria buxi*	Boxwood	Boxwood	Blight

### *Calonectria pseudonaviculata (Cps)* inoculum preparation

Conidia from the culture of *Cps* were used for plant inoculation. Production of conidia was done using fresh potato broth, as described previously [10]. Briefly, before inoculation, conidia were washed off with 30 mL of 0.01% Tween 20 from the mycelial mats that were exposed to fluorescent lights for 3 days after growing in the broth for 4 days in the dark at 23 °C and collected in a 50 mL tube. The suspension was vortexed to break conidia clumps and counted for conidia with a hemocytometer under a microscope (EXC-350 series, ACCU-SCOPE^®^, NY, USA), then diluted with 0.01% Tween 20 to have a concentration of 5 × 10^4^ conidia/mL for inoculation.

### Evaluation of 1021Bp for boxwood blight control

The *in planta* experiment was conducted in the laboratory at 23 °C in a diurnal setting of 16 h dark and 8 h day. Plants of the blight-susceptible boxwood cultivar *Buxus sempervirens* ‘Justin Brouwers’ at age five years were used. They were grown in 2.8-L containers in the greenhouse before being transferred to the laboratory under the conditions described previously [8, 9]. The resuspended cells at 10^8^ to 10^9^ CFU in 0.01% Tween and the CFS from the same concentration of the cell culture were used for plant pretreatment at 1 and 7 days before inoculation (dbi) with *Cps* conidial suspensions at 5 × 10^4^. Additionally, the 18-h cell culture, including the cells and CFS of 1021Bp, was used for plant pretreatment at 1, 2, 3, 6, or 11 weeks (wbi) before inoculation with *Cps*. In these experiments, NB was used as the control of CFS and the cell culture, and 0.01% Tween 20 was used as the control of resuspended cells. Individual experiments were set up for each treatment time, in which three replicate container plants were used for a treatment. After pretreatments, a completely randomized experimental design was used to arrange the container plants in plastic boxes for inoculation with *Cps* inoculum. Both bacterial pretreatments and inoculation with *Cps* were done on the plant canopy by hand spray until runoff. To maintain the moisture facilitating bacterial cell hydration or *Cps* conidia germination and plant infection, treated/inoculated plants were placed in closed large plastic bins within the first 24 h of the pretreatment or inoculation. Each experiment containing three container plants per treatment was conducted twice.

The evaluation of 1021Bp for boxwood blight control was done 7 days after inoculation, when leaf blight symptoms were apparent. The performance was assessed with resuspended bacterial cells and CFS as well as the whole culture without separating cells and CFS. In the first case with resuspended cell and CFS, the total plant leaves and the infected plant leaves, including those that were defoliated, were counted to calculate the leaf infection rate. In the second case with the whole culture, disease severity was estimated using a 1–10 scale representing ascent percentage by 10, as was done in a field experiment [30]: 1 = 1–10%, 2 = 11–20%,. 10 = 91–100% leaves blighted. To be consistent, these disease severity rating data were converted to percentage of leaves blighted at each pretreatment lead time using the formula: (scale level × 10) – 5 before statistical analysis.

### 1021Bp identification

DNA of 1021Bp cells was extracted using the Quick-DNA™ Fungal/Bacterial Miniprep Kit (Zymo Research, CA, USA) and amplified with the universal primers 27 F and 1410R for the 16 S rRNA gene through PCR as described previously [31]. The amplicon was sequenced at Eton Bioscience (Research Triangle Park, NC, USA), and the processed sequence containing a large part of the 16 S rRNA gene was blasted against the GenBank repository at http://blast.ncbi.nlm.nih.gov and deposited into GenBank with Accession: SUB12930864 1021BPseq OQ565619. Colony morphology was compared with the morphology of the taxon that produced the most significant alignment.

### Statistical analyses

All presented data are means of replicates from at least two experiments, expressed as mean ± standard error (SE). Analysis of variance was conducted using the Statistical Analysis Software (SAS Institute, Cary, NC, USA) after log_10_ transformation of the disease assessment data - % leaves blighted. Treatment means were separated according to the least significant difference (LSD) test at *P* = 0.05.

## Results

### Plant growth promotion (PGP)

**Fig. 1:**
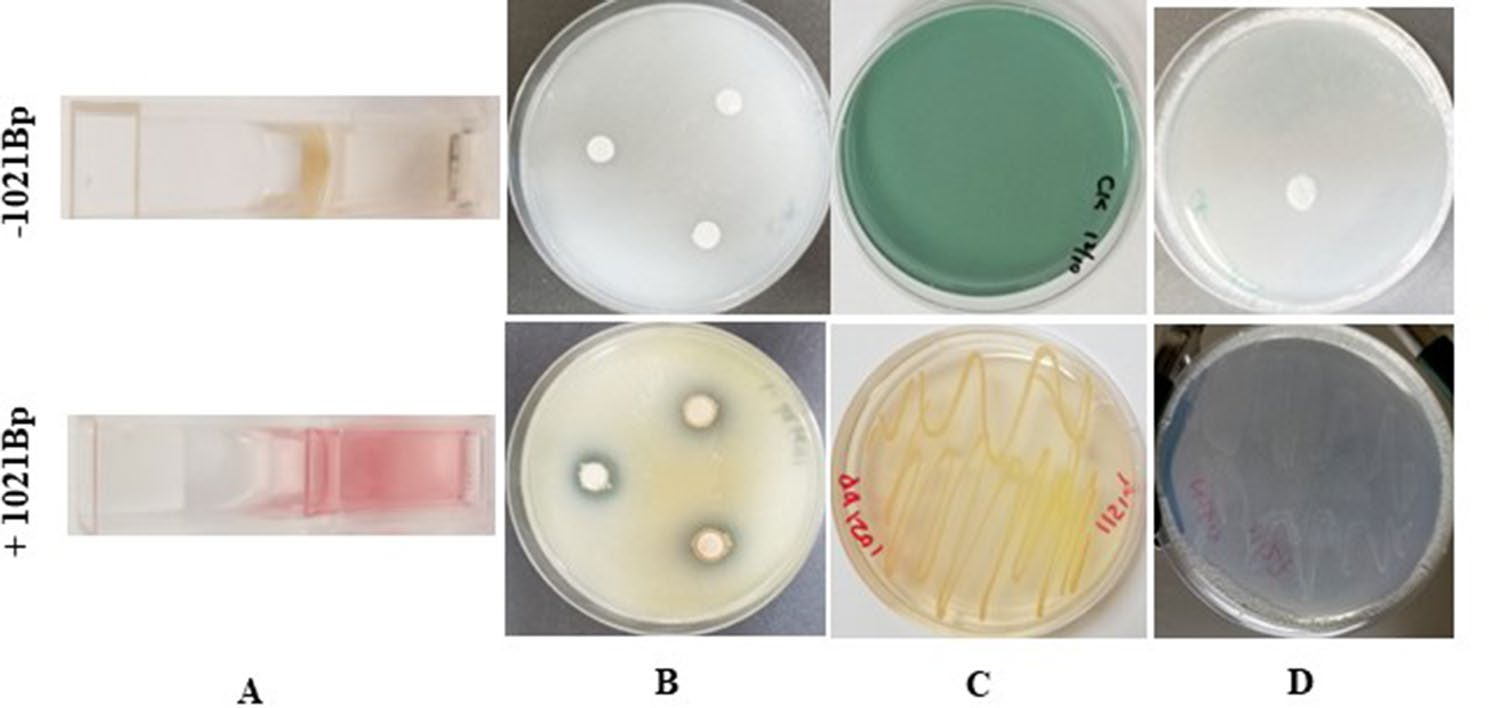
Plant growth promotion (PGP) traits of *Pseudomonas* sp. 1021Bp (bottom) compared with the control (top). IAA production (**A**), phosphate solubilization, (**B**) as indicated by a halo around the discs, siderophore production (**C**) as shown by blue discoloration of the blue medium, and N2 fixation (**D**)

Four PGP characteristics, IAA production, nitrogen fixation, phosphate solubilization, and siderophore making, were detected for 1021Bp. The strain produced IAA in NB culture at 17.67 (± 1.08) µg/mL after 72 h (Fig. 1A). For phosphate solubilization, a colorimetrical assay showed that 1021Bp solubilized 189.3 (± 19.6) parts per million (ppm) of insoluble phosphate in 7 days. This phosphate solubilization ability was also shown by the plate assay. Clear halos appeared around the discs with a bacterial culture but not around the control discs without the 1021Bp after two weeks of disc placement in the media (Fig. 1B). In the plate assay, 1021Bp led to total discoloration of the blue agar medium that was streaked with the bacterial culture in 24 h, indicating its strong positive activity for siderophore production (Fig. 1C). 1021Bp also grew moderately on the nitrogen-free medium, as shown 4 days after streaking (Fig. 1D).

### Fungal growth suppression

**Fig. 2:**
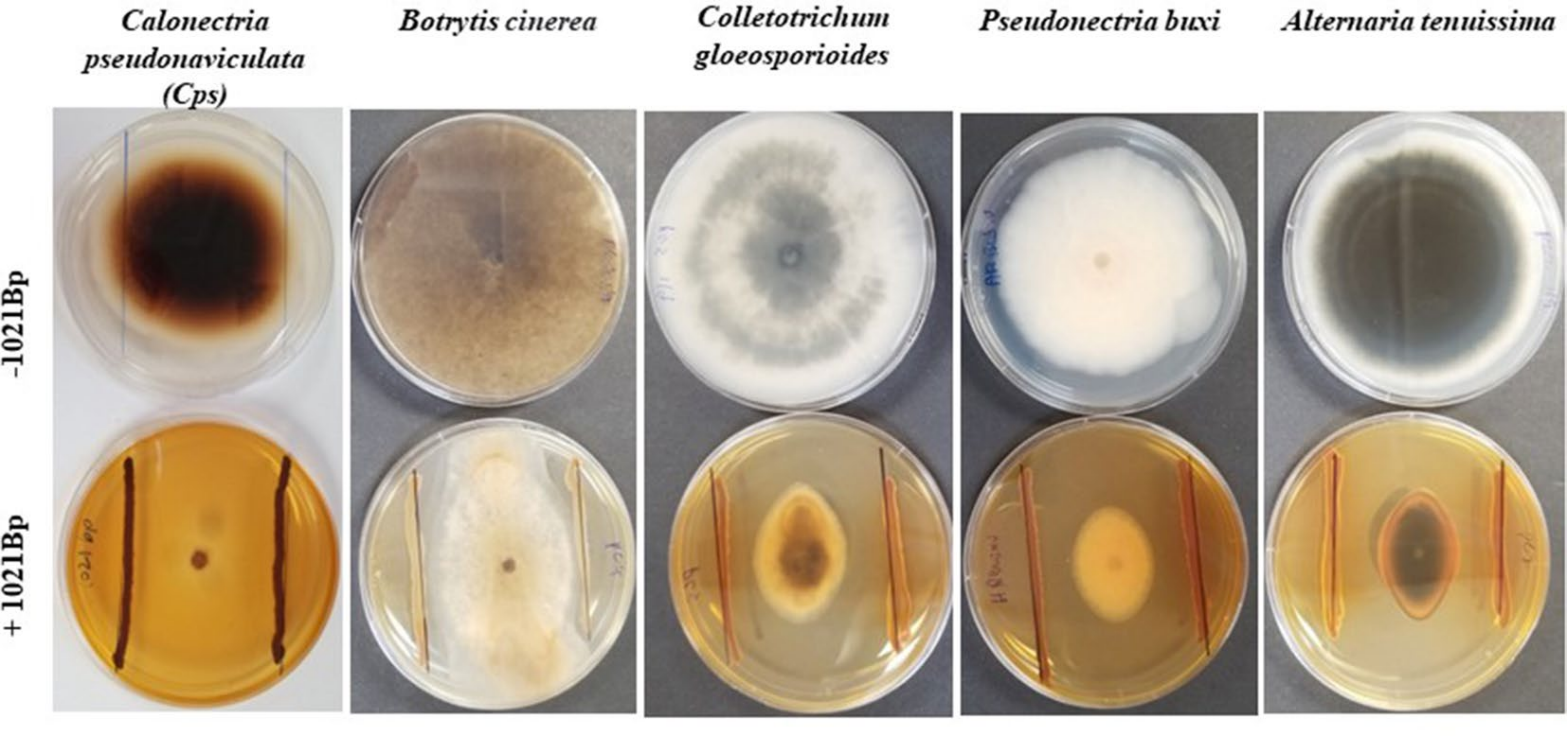
Antagonistic interaction between *Pseudomonas* sp.1021Bp and selected phytopathogenic fungi. Pictures were taken two weeks after a fungal culture plug was placed in the center with (bottom) or without 1021Bp (top) on both sides

1021Bp significantly suppressed the growth of all five fungal pathogens in the dual culture assay (Table 1). The P value (T < = t) two-tail in the T-test between the treatments with and without 1021Bp at both 5- and 10-days post culturing was all less than 0.0001. Among the pathogens, *Cps* was mostly suppressed, followed by *A. tenuissima*, *C. gloeosporioides*, and *P. buxi* (Table 2; Fig. 2). *Botrytis cinerea* was the least suppressed. It grew so fast that it reached the edge of the medium plates by the 5th day after its mycelial plugs were placed.

**Table 2:** Growth suppression of selected fungal plant pathogens by 1021Bp in the dual culture assay

Pathogen	Suppression (%)*	
	5 dpc	10 dpc
*A. tenuissima*	52.8	73.4
*B. cinerea*	48.4	50.8
*Cps*	100	93.4
*C. gloeosporioides*	53.4	72.2
*P. buxi*	25.8	72.8

*n = 6; dpc depicts days post dual culturing

### Boxwood blight control

**Fig. 3:**
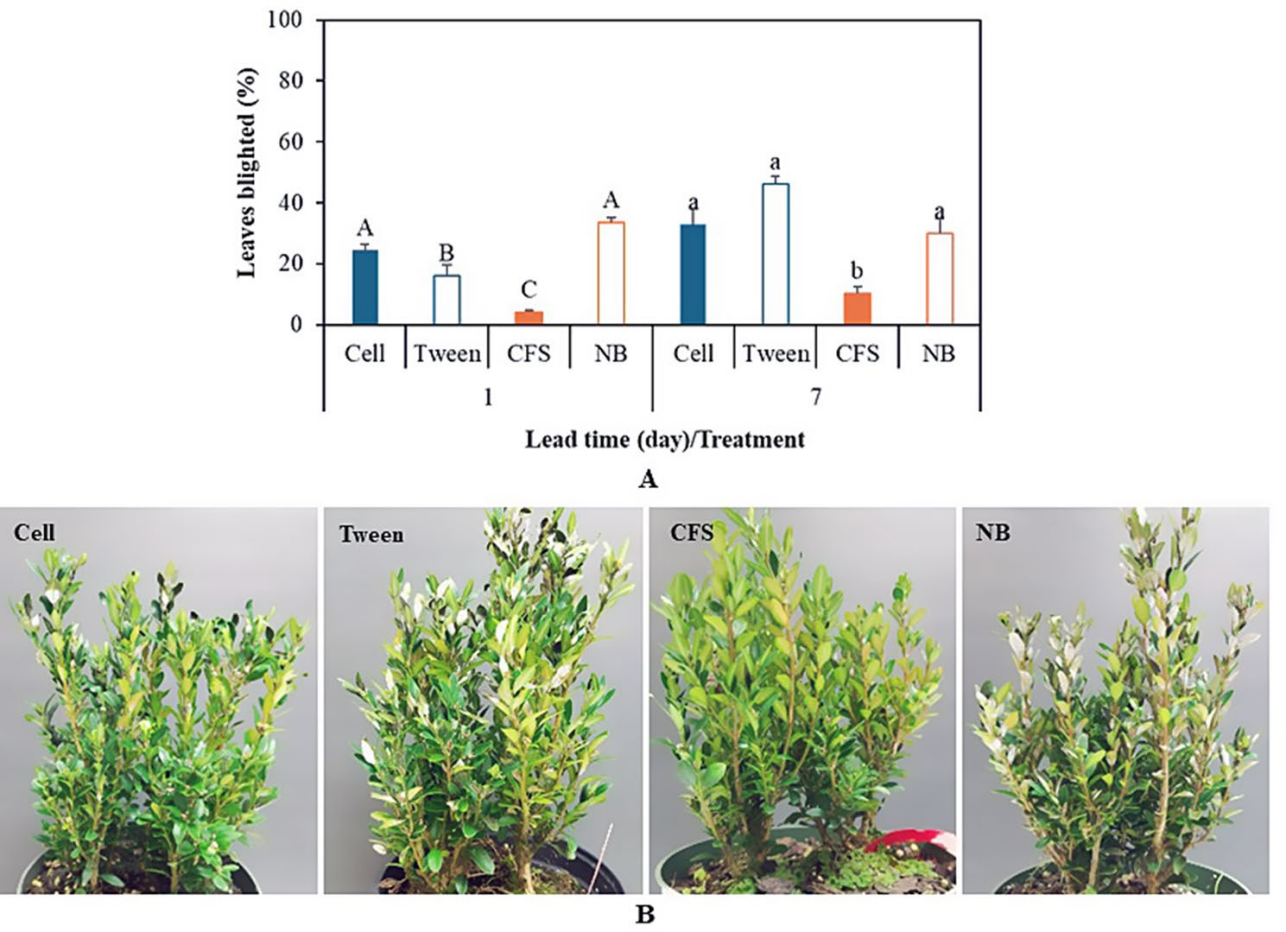
Leaf infection of *Buxus sempervirens* ‘Justin Brouwers’ by *Calonectria pseudonaviculata* (*Cps*) among the four treatments. **A** Infection rates at two lead times of pretreatment prior to inoculation with *Cps*. Each column is the mean of six replicates in two experiments and topped with a standard error bar and letter. Uppercase letters are for treatment one day before inoculation (dbi) while lowercase letters are for treatment at 7 dbi. Columns that share a letter within each lead time did not differ according to the least significant difference (LSD) at *P* = 0.05. Cell depicts resuspended 1021Bp bacterial cells at 10^8–9^/mL 0.01% Tween 20; Tween depicts 0.01% Tween 20 without bacterial cell; CFS depicts cell-free supernatant of bacterial culture in nutrient broth (NB); NB depicts nutrient broth media only. **B** Blight symptoms among the treatments at 7 dbi

CFS of 1021Bp performed the best with 4.2% leaves blighted at 1 dbi, reducing boxwood blight by 87.4% when compared to that of its control – NB which had 33.4% leaves blighted (Fig. 3). In contrast, the resuspended bacterial cells did not perform well. The percentage of blighted leaves from the treatment was significantly higher than that from its control, Tween (Fig. 3). At 7 dbi, CFS consistently performed well, resulting in an efficacy of 65.8% compared to its control. The results indicated strong boxwood blight suppression by the metabolites of the bacterium and the impact of Tween 20 on the bacterial cells.

**Fig. 4:**
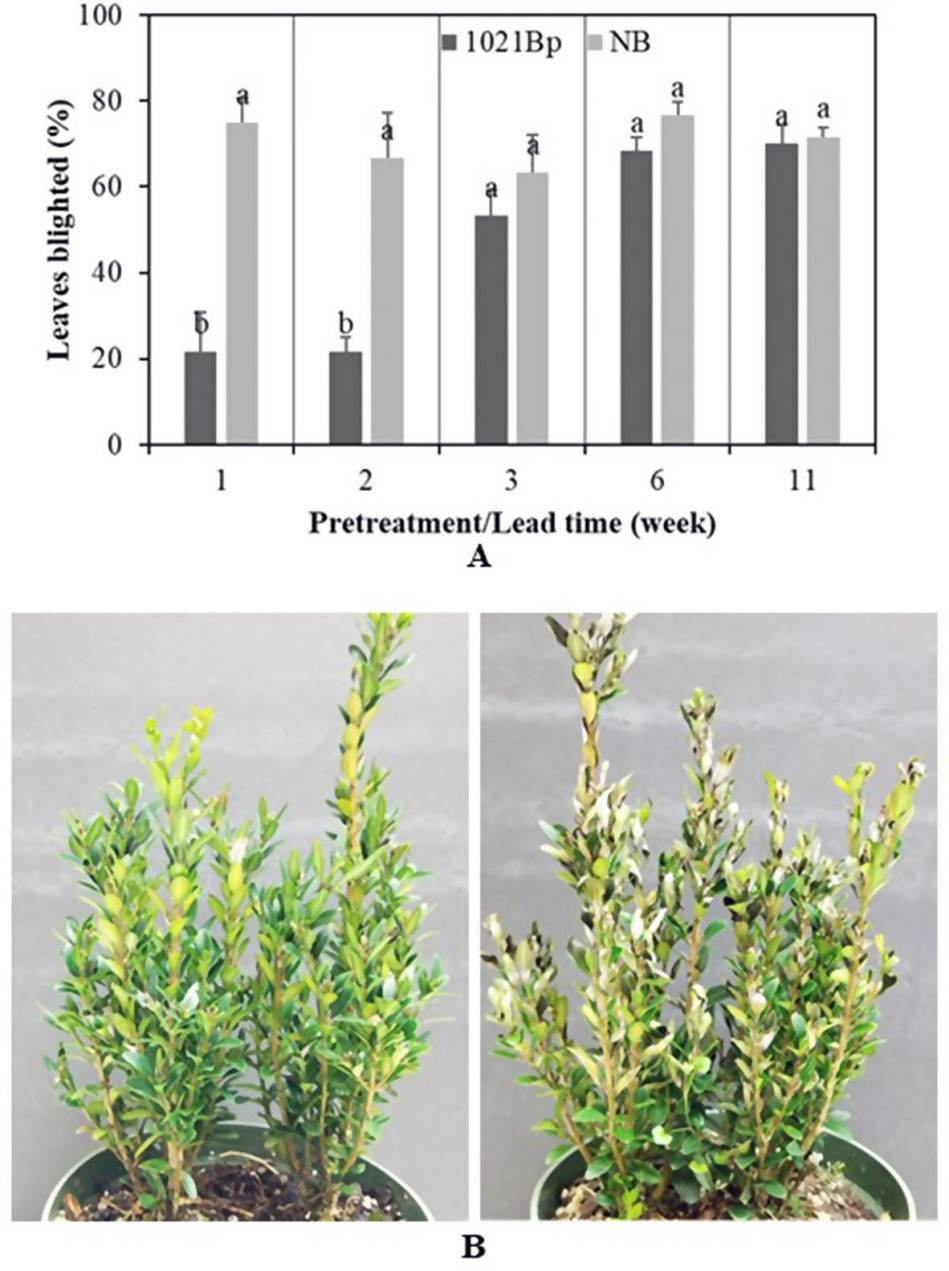
Blight severity of *Buxus sempervirens* ‘Justin Brouwers’ plants pretreated with and without 1021Bp culture at 10^8–9^ cells/mL. **A** Boxwood blight severity at five lead times of pretreatment prior to inoculation with *Calonectria pseudonaviculata*. Each column is the mean of six replicates in two experiments and is topped with a standard error bar. Columns topped with different letters within each of the five lead times (1, 2, 3, 6, and 11 weeks) differed according to the least significant difference at *P* = 0.05. NB depicts nutrient broth media without 1021Bp culture. **B** Blight symptoms developed on boxwood plants pretreated with (left) and without 1021Bp culture (right) one week after inoculation

To eliminate Tween 20’s effect, the broth culture of 1021Bp was used with the control NB in other experiments with additional pretreatment lead time points. Treatments at 1 and 2 weeks before pathogen inoculation (wbi) reduced disease by 71.1% and 67.5%, respectively (Fig. 4). The reduction had a good overlap with the result with CFS (66%) at 7 dbi in the experiment separating bacterium metabolites and cells. However, the blight control by the cell culture at a lead time of 3 wbi or longer was insignificant (Fig. 4).

### Identification of 1021Bp

Based on the results from the 16 S rDNA sequence blast, the closest relative of 1021Bp was *P. eucalypticola*. Among the 100 selected compared sequences producing significant alignments, 1021Bp had the highest match with *P. eucalypticola* NP-1 [19], with a sequence coverage of 99%, an E value of 0, and an identity of over 99.72% (Additional file 2). There was another high match, 99.51% identity with *P. fluorescens* P13 [32] (Additional file 2), which is consistent with *P. eucalypticola* NP-1 that has *P. fluorescens* lineage [19]. 1021Bp also produced large tan colonies on PDA (Additional file 1) and the pigment of streaks between the target fungi was brown (Fig. 1), similar to that described for *P. eucalypticola* NP-1 [19].

## Discussion

Metabolites are great contributors to the in vitro and *in planta* performance of 1021Bp, which is beneficial for plant health. In the in vitro assays, 1021Bp demonstrated features of PGP that have not been studied for its close relative *P. eucalypticola* NP-1 and many members of *P. fluorescens* in the same genetic lineage [20–22]. These traits include IAA, siderophore production, phosphate solubilization, and nitrogen fixation. Specifically, 1021Bp is an excellent auxin producer. It yielded IAA almost five times as much as that produced by another boxwood endophytic bacterium, SSG belonging to *Burkholderia* (3.74 µg/mL) [25]. In the dual culture assay, 1021Bp also showed antifungal activity with its diffused metabolites, suppressing all five test fungal pathogens that have not been included in the report of *P. eucalypticola* NP-1. Great growth suppression was shown not only on two important boxwood pathogens but also on the causal agents of gray mold and anthracnose diseases. The suppression of *B. cinerea* appeared weaker, which may be attributed to the fast growth of the pathogen, as shown by the little difference in suppression rated between five and ten days of the dual culture assay (Table 1). Thus, for gray mold control, pretreatment with 1021Bp is necessary to eliminate growth competition and allow immediate contact with the bacterial metabolites.

The contribution of metabolites was also demonstrated in the *in-planta* assay with 1021Bp CFS which performed significantly better than the resuspended cells when used to protect plants from boxwood blight. This performance is in contrast to that of other previously reported potent endophytic biocontrol agents, SSG [8] and *P. lactis* SW [9]. In the latter scenario, the resuspended bacterial cells performed better or were more effective than their CFS [8, 9, 18]. This difference between the endophytes may be associated with their origins. 1021Bp originated from the microbiome in noninoculated leaf tissue with *Cps*, while the other two originated from the microbiome in inoculated but symptoms-reversed leaf tissue. The latter had been challenged with the pathogens resuspended in Tween 20 before isolation, thus being more tolerant of Tween 20 than 1021Bp when the same condition is present again.

Tween 20 as a nonionic surfactant has been reported to inhibit biofilm formation by *E. coli* at the interfaces of air-liquid and solid-liquid, although it does not impede growth or inhibit curli production at concentrations less than or equal to 0.01% [33]. We used a concentration of Tween 20 at 0.01%, but it is not clear whether the concentration may prevent the cells of 1021Bp from forming a network that can support shear stress or even affect their viability. However, as shown by more infection with the treatment at 1 dbi than 7 dbi in the plant protection experiments (Fig. 3), Tween 20 used in this study likely made such impacts on the bacterial cells. Tween 20 was used in both the pretreatment and inoculation. A shorter interval between the pretreatment and inoculation may be more influential due to double exposure to Tween 20 in a short time. Nevertheless, directly using the cell culture or resuspending the cells in nutrients or media without nonionic surfactants may facilitate cell activity or the performance of 1021Bp in application.

In short, 1021Bp is a potential biofertilizer and biofungicide because of its traits for PGP, broad-spectrum antifungal activity, and high control efficacy on boxwood blight. Specifically, it may have few medical concerns due to not only its plant origin but also its genetic lineage as a member of *P. fluorescens* that is usually non-pathogenic. However, as some members of the genus have been shown to be human opportunistic pathogens, the safety of 1021Bp for humans and other animals remains to be determined. 1021Bp, except for its host difference, is very similar to NP-1, the type strain of *P. eucalypticola* found in *E. dunnii* leaves [19], in sequence, pigmentation, and antifungal activity. Therefore, it is very likely a new member of *P. eucalypticola* that may be widespread as a foliage endophyte in the plant kingdom. However, to confirm its identity, a fatty acid composition assay and full genome sequencing are warranted.

## Electronic supplementary material

Below is the link to the electronic supplementary material.

Supplementary Material 1: Isolation plate of 1021Bp. Three days after a PDA plate spread with the extract of surface sterilized leaves of boxwood (*Buxus sempervirens* ‘Suffruticosa’), one of two large tan colonies (circled in blue) was selected from grown bacterial colonies for subculture.

Supplementary Material 2: Screen shot of blasting 1021Bp 16S rDNA sequence against sequences that produce significant aliments.

## Data Availability

The 16 S rDNA of 1021Bp sequence was deposited into GenBank with Accession: SUB12930864 1021BPseq OQ56561. The datasets analyzed during the current study are available from the corresponding author on reasonable request.
