# Supplementary figures and images for: Evaluation of 1021Bp, a close relative of Pseudomonas eucalypticola, for potential of plant growth promotion, fungal pathogen suppression and boxwood blight control
Source: BMC Microbiol. 2024 Sep 14;24:346. doi: 10.1186/s12866-024-03497-w (PMC11401285; doi:10.1186/s12866-024-03497-w)

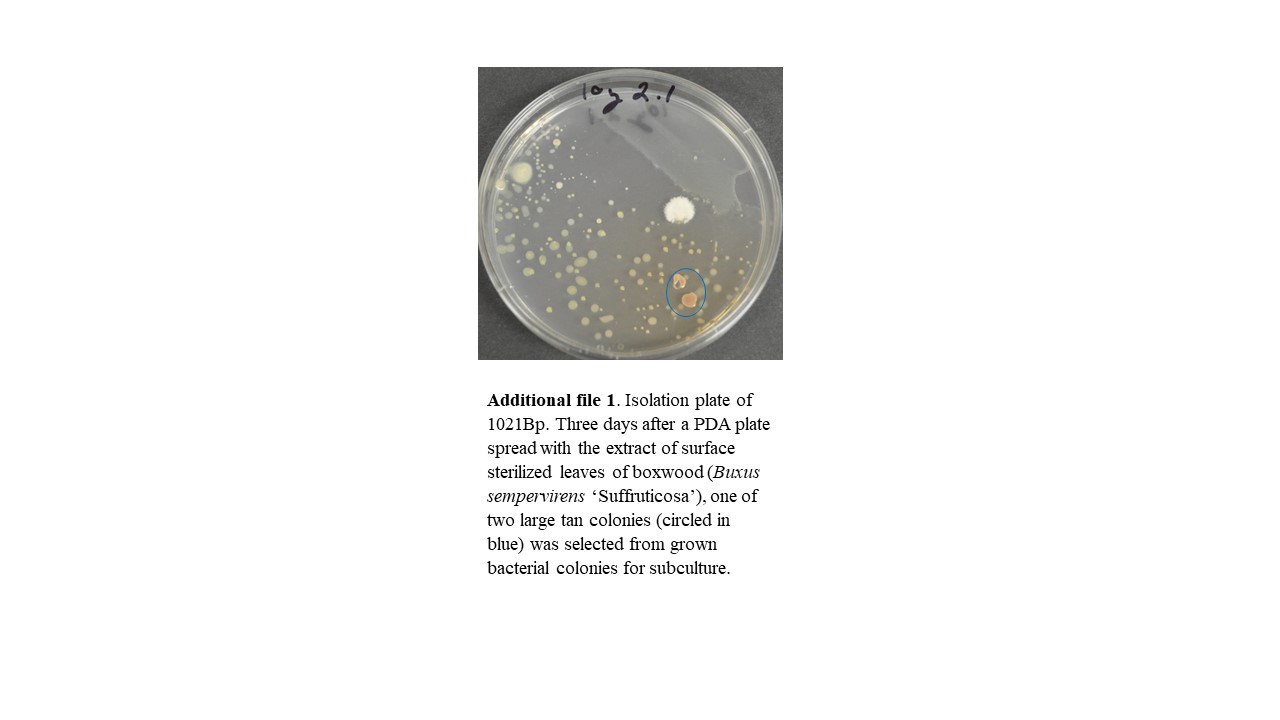

Supplement: Supplementary file 1 — Supplementary Material 1: Isolation plate of 1021Bp. Three days after a PDA plate spread with the extract of surface sterilized leaves of boxwood (Buxus sempervirens ‘Suffruticosa’), one of two large tan colonies (circled in blue) was selected from grown bacterial colonies for subculture. [file 12866_2024_3497_MOESM1_ESM.jpg]

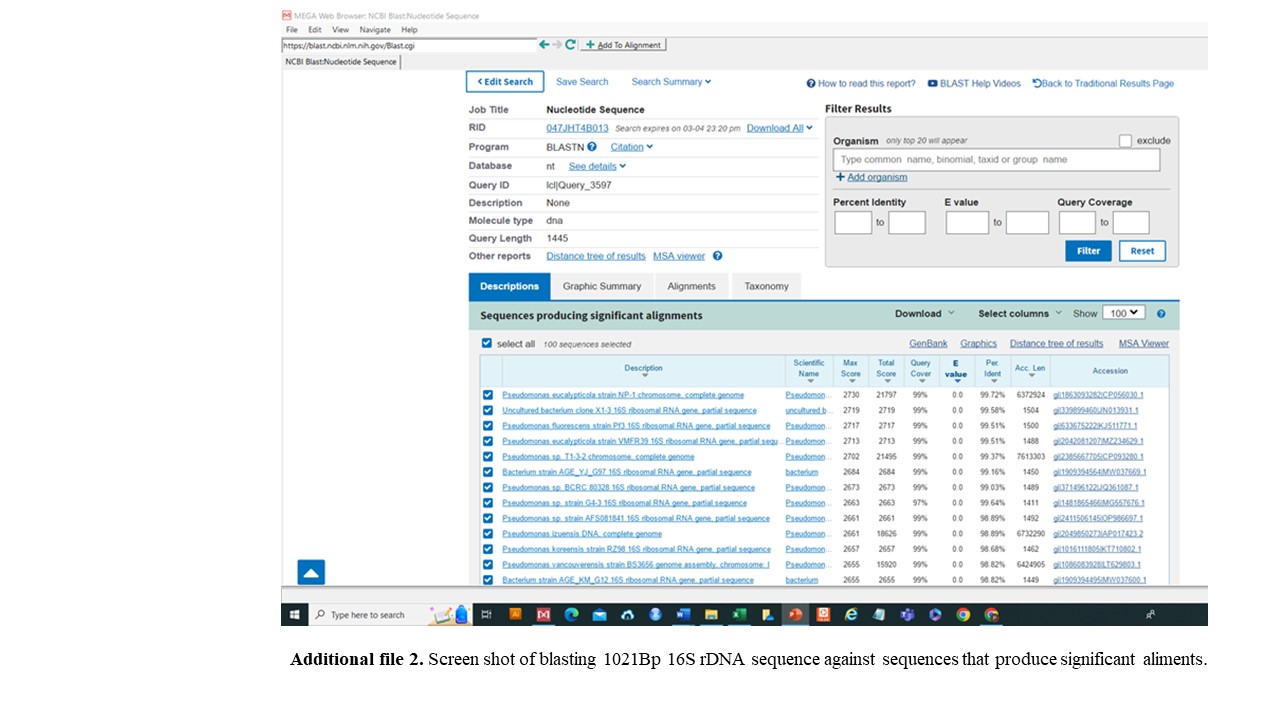

Supplement: Supplementary file 2 — Supplementary Material 2: Screen shot of blasting 1021Bp 16S rDNA sequence against sequences that produce significant aliments. [file 12866_2024_3497_MOESM2_ESM.jpg]
